# Editorial: Data-driven vaccine design for microbial-associated diseases

**DOI:** 10.3389/fimmu.2025.1749184

**Published:** 2026-01-15

**Authors:** Gurudeeban Selvaraj, Satyavani Kaliamurthi, Dongqing Wei

**Affiliations:** 1 Department of Medical Biotechnology, Faculty of Interdisciplinary Studies, Aarupadai Veedu Medical College and Hospital (AVMC&H), Vinayaka Mission Research Foundation (Deemed to be University), Puducherry, India; 2 Centre for Research in Molecular Modeling (CERMM), Department of Chemistry and Biochemistry, Concordia University, Montreal, QC, Canada; 3 Department of Biomaterials, Saveetha Dental College and Hospitals, Saveetha Institute of Medical and Technical Sciences, Chennai, India; 4 State Key Laboratory of Microbial Metabolism, Joint International Research Laboratory of Metabolic & Developmental Sciences and School of Life Sciences and Biotechnology, Shanghai Jiao Tong University, Shanghai, China; 5 Qihe Laboratory, Qishui Guang East, Qibin District, Hebi, Henan, China; 6 Zhongjing Research and Industrialization Institute of Chinese Medicine, Nanyang, China

**Keywords:** artificial intelligence, drug delivery system, gut microbiota, immunoinformatics, multi-epitope vaccine, structural modeling, tuberculosis, Zika virus

Vaccinology is rapidly evolving, driven by the convergence of genomics, immunoinformatics, and artificial intelligence (AI). As infectious diseases continue to challenge global health whether through re-emerging bacterial pathogens, rapidly evolving viruses, or opportunistic microbiota computational tools are becoming central to vaccine discovery. This Research Topic brings together ten diverse yet thematically connected studies that collectively demonstrate how modern vaccinology is shifting toward precision, integration, and predictive modeling. Taken together, these studies illuminate the future direction of the field, data-driven, multi-targeted, and strategically optimized vaccine design.

To begin with, the long-standing shortcomings of the Bacillus Calmette-Guérin (BCG) vaccine in preventing adult pulmonary tuberculosis highlight the urgent need for improved alternatives. In this Research Topic, one study takes a rational genetic approach by creating progressively attenuated *M. tuberculosis* H37Rv strains. By examining macrophage transcriptomic responses to these engineered strains, the authors reveal strong activation of immune pathways including nuclear factor kappa B (NF-κB), tumor necrosis factor (TNF), chemokine signaling, and notably interleukin-17 (IL-17) signaling. Importantly, this upregulation across all vaccine strains suggests a capacity to elicit robust mucosal immunity, thus providing a promising foundation for next-generation TB vaccines (Veerapandian et al.). This work also exemplifies how integrating pathogen genomics with host response profiling can accelerate rational vaccine design.

Building on the theme of precision, the second study focuses on cervical cancer–associated high-risk *human papillomavirus* (HPV) subtypes (Cai et al.). Although current prophylactic vaccines provide broad protection, subtype-specific insights remain essential for refining immunogen design. Through detailed *in silico* profiling of HPV-31 and HPV-52 E6/E7 proteins, the authors identify physicochemical properties, dominant B- and T-cell epitopes, and structural determinants of immunogenicity. These findings not only deepen our understanding of oncogenic HPV variants but also pave the way for subtype-tailored vaccine approaches.

Continuing with mycobacterial pathogens, another study addresses the rising burden of non-tuberculous mycobacteria. By analyzing complete genomes from *M. avium*, *M. intracellulare*, and *M. colombiense*, the authors design a multi-epitope vaccine based on conserved regions of the antigen 85 family. Furthermore, population coverage analysis ensures relevance across African populations, while immune simulations predict strong humoral and cellular responses (Kashiri et al.). Consequently, this cross-species construct represents a significant step toward broad-spectrum mycobacterial immunization strategies.

Similarly, the challenge of human immunodeficiency virus (HIV) vaccine development stems largely from viral variability. In this Research Topic, a study explores a dual strategy: mapping immunodominant epitopes and introducing targeted mutations to enhance recognition across HIV subtype C variants (Kumar Mishra et al.). Structural modeling, TLR3 docking, and long-timescale molecular dynamics simulations collectively demonstrate stable vaccine-receptor interactions. In addition, strong predicted immunoglobulin responses and favorable codon adaptation highlight its translational potential. Thus, this work underscores how rational mutation of epitopes may help overcome viral diversity.

Transitioning to viral encephalitides, one study presents a refined multi-epitope subunit vaccine targeting SLEV. Unlike earlier efforts that focused solely on the E protein, this work incorporates membrane protein M and anchored capsid protein anchC, thereby broadening antigenic coverage. The resulting constructs exhibits high structural stability and strong TLR-4 binding, and immune simulations further indicate robust immunogenicity (Ramalingam et al.). Hence, this expanded antigen strategy showcases how multi-protein approaches can enhance vaccine efficacy against complex RNA viruses.

Expanding beyond classical pathogens, another study examines *Ruminococcus gnavus*, a gut pathobiont implicated in inflammatory bowel disease. Through subtractive proteomics, the authors identify two key virulent proteins and construct a multi-epitope vaccine showing strong TLR4 interaction and structural stability (Dingding et al.). Although experimental validation remains necessary, this work importantly demonstrates how vaccinology can be extended to microbiota-associated diseases, potentially transforming future therapeutic approaches for chronic inflammatory conditions.

In addition, the Research Topic features a comprehensive computational pipeline for designing multi-epitope vaccines against human respiratory syncytial virus (hRSV). By mining conserved regions of F and G glycoproteins and evaluating antigenicity, allergenicity, and structural features, the authors identify promising candidates with strong docking affinity for TLR1 and TLR4 (Alnajran et al.). Coupled with immune simulations predicting high IgG, IgM, IL-2, and IFN-γ levels, the work offers a compelling alternative to the limited RSV vaccines currently available for older adults.

Meanwhile, the Zika virus continues to pose a threat in Asia, particularly India. This study identifies novel linear and conformational epitopes in both envelope and NS1 proteins of circulating Indian strains and evaluates their interactions with potent neutralizing antibodies (Roy et al.). The discovery of epitopes capable of strong engagement with monoclonal antibodies such as ZV-67 and Z3L1 provides critical information for developing next-generation, lineage-specific Zika vaccines.

Further reinforcing the theme of genomic integration, another study conducts a pangenome analysis to identify conserved virulence determinants in *Pseudomonas aeruginosa* (Elavarasu and K). Prioritizing the outer membrane protein *Lpt*F, the authors design a multi-epitope vaccine with stable TLR interactions and predicted high expression in *E.coli*. Immune simulations additionally indicate strong adaptive responses, including memory B-cell and T-cell activation. Therefore, this construct holds promise for addressing antibiotic-resistant *P. aeruginosa* infections.

Finally, moving from antigens to delivery systems, the Research Topic concludes with an innovative AI-driven framework for optimizing lipid nanoparticles formulations for mRNA vaccines (Di Salvatore et al.). By generating synthetic transcriptomic datasets to emulate tissue-specific responses, and integrating random forest modeling with a genetic algorithm, the authors identify nanoparticles designs with minimized off-target immune activation. As a result, this purely in silico pipeline offers a paradigm shift toward safer and more targeted mRNA vaccine delivery strategies.

These ten studies collectively showcase the transformative impact of computational biology in rational vaccine design. By integrating structural biology, immunoinformatics, molecular docking, AI, and immune simulations, each contribution extends the frontiers of vaccinology beyond traditional paradigms. Moreover, the spectrum of pathogens addressed from bacteria and viruses to gut microbiota demonstrates the versatility and applicability of these approaches across disease domains. As we face future outbreaks and emerging antimicrobial resistance, these studies lay the groundwork for agile, intelligent, and personalized vaccine development. I extend my gratitude to all contributing authors for their innovative efforts, and I am confident that this body of work will inspire further translational and experimental endeavors in infectious disease research.

